# Song Diversity Predicts the Viability of Fragmented Bird Populations

**DOI:** 10.1371/journal.pone.0001822

**Published:** 2008-03-19

**Authors:** Paola Laiolo, Matthias Vögeli, David Serrano, José L. Tella

**Affiliations:** 1 Estación Biológica de Doñana (CSIC), Sevilla, Spain; 2 Instituto Cantábrico de Biodiversidad (CSIC, UO, PA), Universidad de Oviedo, Oviedo, Spain; University of Helsinki, Finland

## Abstract

In the global scenario of increasing habitat fragmentation, finding appropriate indicators of population viability is a priority for conservation. We explored the potential of learned behaviours, specifically acoustic signals, to predict the persistence over time of fragmented bird populations. We found an association between male song diversity and the annual rate of population change, population productivity and population size, resulting in birds singing poor repertoires in populations more prone to extinction. This is the first demonstration that population viability can be predicted by a cultural trait (acquired via social learning). Our results emphasise that cultural attributes can reflect not only individual-level characteristics, but also the emergent population-level properties. This opens the way to the study of animal cultural diversity in the increasingly common human-altered landscapes.

## Introduction

In the face of widespread anthropogenic habitat destruction, small populations are becoming a common reality and a paradigm for conservation biology [1]. Incorporating the study of cultural attributes in a conservation context has been recently suggested as a novel way to pinpoint the diversity and status of animal populations [2]. The song of passerine birds is probably one of the best studied examples of cultural traits in non-human animals. This conspicuous and exaggerated display is shaped by sexual selection in the context of male-male competition or mate attraction [3]. Several studies have ascertained the honesty of song features at the level of the individual, the target of selective forces and of behavioural research [4], [5]. Via their condition dependence at the individual level, costly male sexual traits have the potential to become good candidates for viability at the level above, that of the population [6], [7]. In spite of this premise, the relationship between birdsong and population properties, such as viability, has received poor attention so far, and widespread species with abundant populations were the target of most studies. Notably, contrasting results with respect to theoretical expectations were obtained when studying the effects of sexual selection on signalling in small populations [8], [9]. In the latter studies, emergent population features (such as population size, density, and migratory status) conditioned song output together with individual determinants, and these effects can emerge only below determinate thresholds [10], [11]. Such results suggest an unexplored, potential link between birdsong characteristics and population properties.

In this study, we focus on social and demographic correlates of song performance in the meta-population system of the Dupont's lark *Chersophilus duponti*, a threatened passerine that is experiencing extinction events in the smallest nuclei in the Iberian Peninsula [12]. Recent studies revealed that the communication systems of this songbird are disrupted by habitat fragmentation which reduced population sizes and created barriers to individual movements and cultural transmission [11], [13], [14]. Here, we investigated two parameters of singing performance that appear more than others to be reliable signals of individual quality in many oscine species: song rate and song diversity (repertoire size) [15]. These traits are though to be costly because of their correlation with brain size and nutritional condition during learning (song repertoire size), and their oxygen consumption and energy expenditure (song rate) [16]. We analyse the relationship between these characters and parameters related to population social milieu and viability (number of neighbour territories, male population size, productivity, annual rate of population change), to test whether a cultural behaviour, birdsong, can reveal the demographic trends of populations, apart from exerting its sexual function among individuals [4].

## Methods

### Study area and Dupont's lark song

The study was carried out in Ebro Valley (northeastern Spain), a 10 500 km^2^ dry plain where patches of steppe vegetation are interspersed in a matrix of prevailing cereal cultivations. Dupont's lark is a typical inhabitant of these natural shrub-steppes and has suffered a recent decline associated to habitat loss and fragmentation [12], [17]. From spring 2004 to spring 2007, we comprehensively surveyed the Dupont's lark metapopulation in Ebro Valley, and recorded vocalizations from most of the populations inhabiting the area (hosting 2 to 48 males each; 1). The fragmented populations of Ebro Valley are experiencing extinction, emigration and immigration events, thus can be well ascribed to a metapopulation system [11], [12]. Populations occupy patches of suitable habitat-islands separated by a sea of agricultural land [14], thus can be easily identified as distinct local nuclei [13].

Songs and territorial calls, the two commonest male vocalizations, were recorded in individual territories by means of a Sony TC-D8 DAT recorder and a Sennheiser ME67 microphone. We also recorded the number of countersinging individuals from neighbour territories, and caller or singer locations by means of a Global Positioning System Garmin eTrex® Navigator. While the song is used in a sexual context during breeding, the territorial call has the function to advertise male identity within territories throughout the year, with peaks in the pre- and post-breeding months (i.e. in February and from September to November) [11], [13], [14]. In this study, we used information from both vocalizations, addressing to the relationship between the song and population viability, and employing acoustic marking with the territorial call (see below) to estimate demographic parameters for population viability analysis.

We recorded good quality-complete song repertoires from 155 males in 19 presently occupied patches (two small populations went extinct during the study and could not be included in PVA, see below). Dupont's lark song includes a variable number of discrete phrases or song types that are shared by neighbouring males during spring disputes (song spectrograms can be found in Laiolo & Tella [11]). We addressed to two parameters of singing performance that can be used in both within and among population comparisons: song diversity and song rate. We did not consider variation in the acoustics or the occurrence of specific song structures because of the great variability in song types among patches, which makes spectrotemporal comparisons among populations useless [13].

As a measure of song diversity, we characterized individual repertoires and classified song types by visually inspecting over 5700 spectrograms, displayed by Avisoft SASLab Pro 3.91 Software (Fast Fourier Transform: sampling frequency 22050 Hz, FFT length 512, time resolution 8.9 msec, frequency resolution 43 Hz, Window Function: Bartlett). The song diversity of an individual equals the number of distinct song types identified in its repertoire. The classification criterion of Laiolo & Tella [13], [14] was followed, with song types categorized blind to assure consistency of classification. Dupont's lark males utter their complete song repertoire from one to several times during song bouts before dawn. We estimated individual song rate as the number of song types uttered per minute from males that uttered at least three times the average individual song repertoire [18], [19].

### The use of territorial calls for the identification of individuals and yearlings

In a previous paper, we demonstrated that the territorial calls from a sample of banded males were individually distinctive and constant over time in their acoustic features, and could be used for individual-based monitoring of Dupont's lark populations [20]. For the present study, we measured spectrotemporal variables from 3756 territorial call types recorded in the field during the study period, and then used discriminant function analysis to calculate Euclidean distances between calls of every pair of recorded males. Individuals recorded in different days were considered the same if their acoustic distance was less than the maximum within-individual distance of banded males [20]. The repeated recording of a given individual over years was considered as equivalent to a recapture. Although 333 different individuals were identified by discriminant analysis, for annual survival analyses we retained data from 208 males recorded at least once in spring.

In many passerines species, vocal learning takes some time and generally comes to an end only shortly before the bird establishes its own territory [21]. In Dupont's lark yearling, the utterance of territorial calls in summer is the way to flag the turf. These calls can be recognized from those of adults by the presence of quavering harmonics and relatively amorphous notes evidenced in spectrograms; their quality improves gradually in a process that lasts less than 30 days and peaks in summer and early autumn (13; Fig. S1). By quantifying the relative proportion of yearlings calling in the whole population of vocalizing males in the summer-autumn period (adults plus young), we obtained an estimate of yearling to adult male ratio. This ratio is commonly used as a surrogate of population productivity when actual reproductive success is difficult to record [22], [23], as it is the case of the secretive Dupont's Lark [14]. In doing this, we are assuming that all the young recorded in a population were born there, since the few immigration events could be effectively detected and discarded as we were able to identify males uttering a foreign dialect (i.e. territorial calls typical of another patch) [11]. These proportions were used to scale the maximum, theoretic productivity values for population viability analyses (see below).

### Survival and population viability analysis

We started from the Cormack–Jolly–Seber model for open populations (24), where apparent survival (φ) and reencounter (*p*) probabilities were time-specific for each population size (classes of <10, 11–30 and >30 individuals). We first assessed the fit of this global model using program U-CARE 2.2.2 [25], and then model structures were implemented in program MARK 4.3 [26]. We used the Akaike's Information Criterion corrected by sample size AICc to select the most parsimonious model (27). Models were ranked on the basis of the differences between the AICc of a given model and the AICc of the highest ranked model (ΔAICc), and on the basis of a measure of the weight of evidence for a model expressing the probability that that model is actually the best one (AICc weight) [28].

Population size in our analyses had little influence on mark–recapture estimators of Dupont's lark survival, and the most probable model included annual variation in survival (differences among the first and the following years) and constancy in recapture rate (Table S1). As reencounter rates were low in 2005 (14%; 95% confidence intervals: 5–32%), we used survival rates as estimated for years 2005 and 2006 (φ = 0.46±0.075 SE) in the following population viability analyses.

The computer program VORTEX [29] was used to simulate the stochastic and deterministic forces affecting the Ebro Valley meta-populations and calculate indices of population viability. Simulations were run 100 times, the time frame was set at 100 years and extinction was defined as only one sex remaining in the population. The values of life-history parameters entered in simulations are provided in Table S2. We run very conservative simulations, and considered similar mortality rates for all age classes and sexes (54%, derived from φ = 0.46 resulting in survival analyses). The number of young per females was calculated as the double of published average clutch size (2.76), since two clutches per years are laid; [30]) corrected by considering the proportion of juveniles to adults in each population (Table S2). We built different scenarios with respect of the percentage of successful breeding females, in order to incorporate in simulations a correction for nesting failure, which is quite high and due to predation in ground-nesting passerines of Spanish steppelands [31]. Percentages of successful breeding females were set at 100, 90, 80, 70, 60, 50, and 40%.

### Correlates of song performance

We first analysed the relationship between song rate and diversity *versus* population viability, expressed as the annual rate of population change (λ). We used λ as it covaried with other population viability indices, such as the exponential rate of increase, the median extinction time and the mean extinction time (0.53<*r*<1.00, all *p*<0.05). We pointed to the average annual rate of population change λ calculated in the seven different simulation scenarios (from 40 to 100% females breeding successfully). To highlight which were the components of population viability that contributed most to song performance, we also analyzed the relationships between song rate and diversity *versus* male population size and productivity (yearling to adult ratio) per patch. We did not enter mortality in the model because survival did not vary among patches, but we considered the number of countersinging individuals (neighbours singing in the same song bout), a parameter related to population social milieu.

We used generalized linear mixed models (GLIMMIX macro in SAS 8e) with a Poisson distribution of error (in the case of song diversity) and a Normal distribution of errors (song rate); population identity entered as a random factor.

## Results

**Figure 1:**
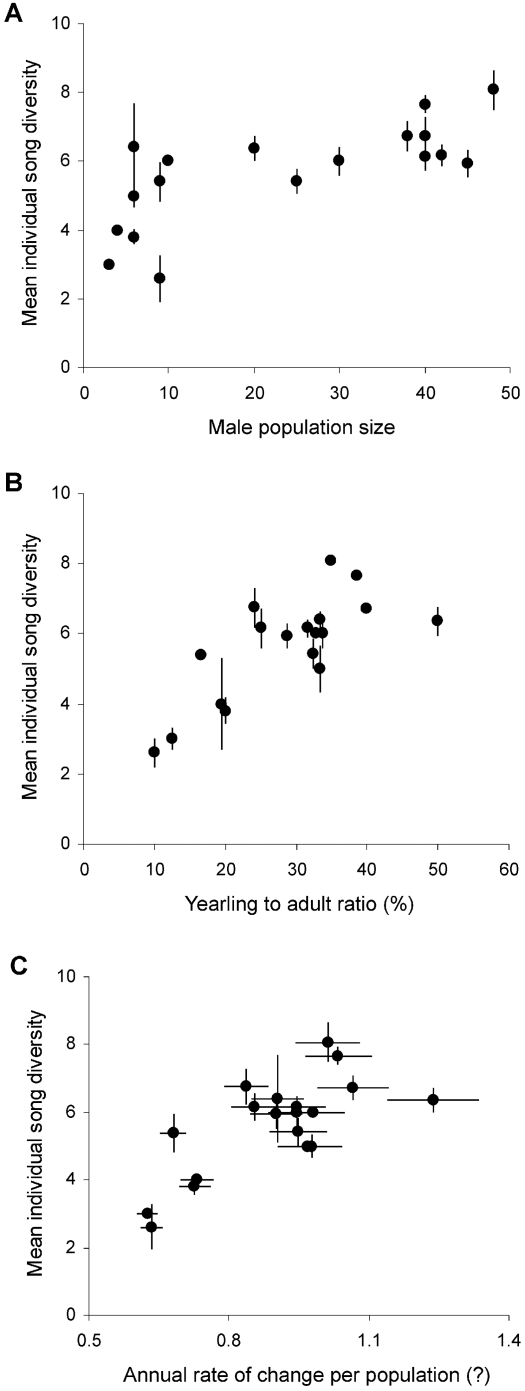
Relationship between mean individual song diversity (±SE) and A) male population size B) patch productivity (yearling to adult ratio), C) population viability (expressed as the mean (±SE) annual rate of population change λ under different scenarios).

We found a significant association between male song diversity and an index of population viability, the annual rate of population change (GLMM: F_1, 136_ = 20.51, P<0.001; Parameter Estimate±SE = 1.25±0.27). Bird populations uttering simpler songs were more prone to extinction (with lowerλ) than populations presenting more complex signalling (Fig. 1).

Song diversity was positively related to population size and population productivity, but was not significantly associated to the number of countersinging males (GLMM with Yearling to adult ratio+Male population size+Number of countersinging males: F_1, 134_ = 14.67, 17.57 and 0.001; P<0.001, <0.001 and = 0.93; Parameter Estimates±SE = 0.008±0.01, 0.002±0.003 and 0.001±0.019 respectively). Therefore, birds from small and poorly productive populations uttered poor repertoires compared to those of large and productive populations (Fig. 1).

The interactions among neighbours had no significant effect on individual song diversity (GLMM: F_1, 136_ = 0.06, P = 0.8), but affected song rate (GLMM: F_1, 136_ = 58.6, P<0.001; Parameter Estimate±SE = 1.68±0.22). Song rate was not significantly associated to population viability, size, or productivity (GLMM: 0.04<F_1, 136_<2.08, 0.15<P<0.84).

## Discussion

By using a new approach that integrated behavioural and cognitive ecology with bird population ecology, we found an association between individual song diversity and an index of population viability, the annual rate of population change. So far, the relationship between behavioural features and population viability has been hypothesised in a few animal species [7], [32], and never using cultural attributes. As mortality did not vary among populations, differences in male population size and productivity explained a large amount of the variation in population viability, and thus strongly affected song diversity (Fig. 1).

Song diversity can thus be viewed as an indicator of population viability, through its relationship with population size, affecting the population social environment, and productivity, depending on individual breeding performance and sexual advertising. A large male number likely contributed to increase the song pool from which individuals picked up their song repertoire through imitative singing, in turn determining the observed increase of individual song pools with male numbers [11]. In support of our hypothesis, we found Dupont's larks singing heterospecific song types in three small populations (<10 males). This species does not imitate other birds as a general rule (contrarily to other lark species; 33), but apparently can shift its strategy to increase song diversity when few conspecific templates are available. To date, there is still limited direct evidence of song diversity erosion in small bird populations [11], possibly because the phenomenon may be only evident at low population sizes (<50 males, Fig. 1B). Effects of population reduction have been indeed demonstrated in a variety of behaviours other than birdsong. Group size decline can disrupt behavioural patterns in some social mammal species [34], and the reduction of speaker numbers is known to threaten the persistence of human languages [35], [36].

A rich repertoire was recorded in populations presenting a large proportion of juveniles, independently of population size. This relationship can result from the sexual nature of song, as reliable indicator of individual fitness [15]. It cannot be excluded that territory quality and breeding time are mediating the above association [37], although we do not expect habitat quality and patch or population size to covary, because bird survival did not depend upon the size of populations. The interactions among neighbours had no significant effect on individual song diversity, but Dupont's lark males sang at faster rates where large clusters of countersinging neighbours were found. In the latter conditions, elevated song rates were possibly associated with escalated interactions among males [13], a phenomenon described also in other passerines [38].

In the absence of definitive data on female choice, we cannot affirm that Dupont's lark females actually use song diversity in making mating decisions, although a multitude of studies are consistent with the idea that large song repertoires are ‘sexy’ [39]. In our study case, females from small populations possibly cannot afford to be choosy on the basis of song traits (as the general song pool of these populations is poor), unless they can sample males from different size patches during dispersal. If females do not leave their natal site because of barriers to dispersal across ecotones [13], [14], a relaxation of sexual selection could occur in small patches with limited competition levels, in turn causing a mismatch between individual quality and sexual signalling [8], [9]. In the long run, this phenomenon could increase the chances of population extinction if the probability of fixing deleterious new mutations increases and that of fixing beneficial mutations decreases as a consequence of reduced female choice [40]. If females can disperse and settle in large patches with appealing songs from local males, the viability of smaller fragments could decrease at an even faster rate, with demographic factors prevailing over genetic ones in extinction processes [41]. Notably, song cues could be also used by dispersing males in their settlement decisions, especially in the absence of direct evidence of habitat suitability [42]. The role of individual choice is indeed crucial to determine whether variation in song diversity plays an active role in population decline or it is a side-effect of it, an alternative we cannot exclude.

This research shows that a population-based approach can complement individual-based studies when attempting to disentangle the causes and consequences of variation in cultural attributes, given that demography may play an important role in social learning processes. In the light of this, studies on cultural attributes on species with small and fragmented populations should be prioritized, because widespread species may respond quite differently to environmental perturbations and thus provide incomplete information on the wide array of natural and human-driven circumstances [5]. On the other hand, our research identifies a cultural trait whose diversity could be used as an early warning signal of population decline (either because of small population size or poor productivity), and provides a novel methodological approach for the monitoring of endangered species. More than 500 songbird species are globally threatened, most of them because of habitat loss and fragmentation in a variety of ecosystems and remote regions [43]. In these conditions, traditional long-term population monitoring is a difficult if not unaffordable task. Given its easily quantifiable nature, we suggest that birdsong could become a good tool to investigate animal cultures within a population ecology context. From a conservation point of view, the diversity of cultural attributes should be incorporated in studies on animal species capable of social learning. Conservation biology will indeed benefit from behavioural research broadening its individual perspective and responding to the social need (and mandate) to preserve species in human-transformed landscapes [44], [45].

## Supporting Information

Figure S1 Spectrograms of territorial calls uttered by a yearling (A) and an adult (B) Dupont's lark male in summer. Yearling calls are characterized by quavering, amorphous notes and repertoire instability; adult calls are repeated in a stereotyped way.
(6.67 MB TIF)

Table S1 Models of survival (φ) and reencounter (p) probabilities for Dupont's lark males at the Ebro valley. Subscripts denote constant (.), time (t), and subpopulation (n) effects. The Cormack-Jolly-Seber model with subpopulation-specific rates in survival and reencounter φ (n * t) p (n * t) fit the data adequately (global test, chi-square = 10.43, d.f. = 10, P = 0.403; one-tailed directional test for transience, Z = 1.63, P = 0.051; signed test for trap-dependence, Z = −0.49, P = 0.63) and was used as the starting point in model selection. We show AICc values, differences in AICc with respect to the top-ranked model (ΔAICc), relative weight of evidence (AICc weight), number of parameters (np), and deviance of each candidate model. Only the 14 most probable models (with highest AICc) are shown.
(0.06 MB DOC)

Table S2 (0.05 MB DOC)
