# Supplementary figures and images for: Song Diversity Predicts the Viability of Fragmented Bird Populations
Source: PLoS One. 2008 Mar 19;3(3):e1822. doi: 10.1371/journal.pone.0001822 (PMC2266806; doi:10.1371/journal.pone.0001822)

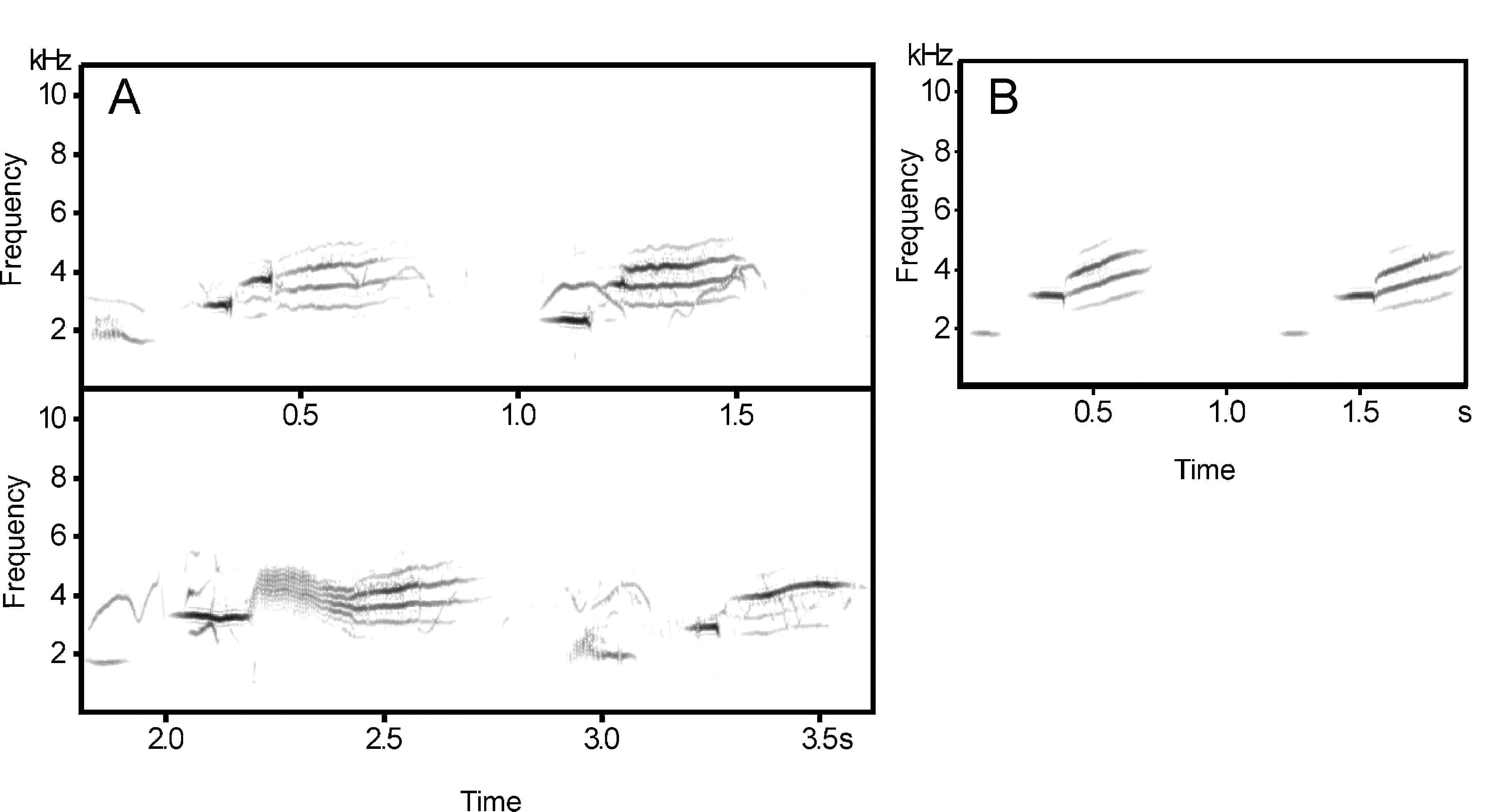

Supplement: Figure S1 — Spectrograms of territorial calls uttered by a yearling (A) and an adult (B) Dupont's lark male in summer. Yearling calls are characterized by quavering, amorphous notes and repertoire instability; adult calls are repeated in a stereotyped way. (6.67 MB TIF) [file pone.0001822.s001.tif]
