# Supplementary material for: Song Diversity Predicts the Viability of Fragmented Bird Populations
Source: PLoS One. 2008 Mar 19;3(3):e1822. doi: 10.1371/journal.pone.0001822 (PMC2266806; doi:10.1371/journal.pone.0001822)
Supplement: Table S1 — Models of survival (φ) and reencounter (p) probabilities for Dupont's lark males at the Ebro valley. Subscripts denote constant (.), time (t), and subpopulation (n) effects. The Cormack-Jolly-Seber model with subpopulation-specific rates in survival and reencounter φ (n * t) p (n * t) fit the data adequately (global test, chi-square = 10.43, d.f. = 10, P = 0.403; one-tailed directional test for transience, Z = 1.63, P = 0.051; signed test for trap-dependence, Z = −0.49, P = 0.63) and was used as the starting point in model selection. We show AICc values, differences in AICc with respect to the top-ranked model (ΔAICc), relative weight of evidence (AICc weight), number of parameters (np), and deviance of each candidate model. Only the 14 most probable models (with highest AICc) are shown. (0.06 MB DOC) [file pone.0001822.s002.doc]

**Tab. 1.** Models of survival (φ) and reencounter (*p*) probabilities for Dupont’s lark males at the Ebro valley. Subscripts denote constant (.), time (*t*), and subpopulation (*n*) effects. The Cormack-Jolly-Seber model with subpopulation-specific rates in survival and reencounter φ (*_n_* * *_t_*) *p* (*_n_* * *_t_*)  fit the data adequately (global test, chi-square = 10.43, d.f. = 10, *P* = 0.403; one-tailed directional test for transience, Z = 1.63, *P* = 0.051; signed test for trap-dependence, Z = -0.49, *P* = 0.63) and was used as the starting point in model selection. We show AICc values, differences in AICc with respect to the top-ranked model (ΔAICc), relative weight of evidence (AICc weight), number of parameters (*np*), and deviance of each candidate model. Only the 14 most probable models (with highest AICc) are shown.

| Model | AICc | ΔAICc | AICc weight | *np* | Deviance |
| --- | --- | --- | --- | --- | --- |
| φ (*_t_*_1(_*_t_*_2=_*_t_*_3))_ *p* (.) | 275.76 | 0.00 | 0.392 | 2 | 36.5 |
| φ (*_t_*_1(_*_t_*_2=_*_t_*_3))_ p (*_t_*_1(_*_t_*_2=_*_t_*_3))_ | 277.71 | 1.95 | 0.148 | 3 | 36.4 |
| φ (*_t_*_1(_*_t_*_2=_*_t_*_3))_ p (*_t_*) | 277.84 | 2.07 | 0.139 | 4 | 34.4 |
| φ (*_t_*) *p* (.) | 277.89 | 2.12 | 0.135 | 4 | 34.5 |
| φ (*_t_*) *p* (*_n_*) | 277.98 | 2.21 | 0.129 | 6 | 30.3 |
| φ (*_t_*) *p* (*_t_*) | 289.94 | 4.17 | 0.049 | 5 | 34.4 |
| φ (.) *p* (.) | 286.51 | 10.74 | 0.002 | 2 | 47.2 |
| φ (*_n_* * *_t_*) *p* (*_n_*) | 286.98 | 11.21 | 0.001 | 12 | 26.1 |
| φ (.)  *p* (*_n_*) | 287.16 | 11.39 | 0.001 | 4 | 43.7 |
| φ (*_n_* * *_t_*) *p* (.) | 287.85 | 12.08 | 0.001 | 10 | 31.5 |
| φ (*_n_*) *p* (*_n_*) | 288.32 | 12.55 | 0.001 | 6 | 40.6 |
| φ (*_n_*) *p* (.) | 288.50 | 12.74 | 0.001 | 4 | 45.1 |
| φ (*_n_* * *_t_*) *p* (*_t_*) | 290.96 | 14.09 | 0.000 | 11 | 31.3 |
| φ (*_n_* * *_t_*) *p* (*_n_* * *_t_*) | 291.42 | 15.71 | 0.000 | 15 | 23.7 |
