# Supplementary material for: Song Diversity Predicts the Viability of Fragmented Bird Populations
Source: PLoS One. 2008 Mar 19;3(3):e1822. doi: 10.1371/journal.pone.0001822 (PMC2266806; doi:10.1371/journal.pone.0001822)
Supplement: Table S2 — (0.05 MB DOC) [file pone.0001822.s003.doc]

**Tab. S2.** Values of life-history parameters used in simulations of population persistence and viability.

| Parameter | Values used | Bibliographic source |
| --- | --- | --- |
| Dispersal | - 10% of dispersing individuals among populations < 15 km apart from each other | [1] |
| Reproductive system | - Monogamous - Age of first breeding: 1 yr for both males and females - Maximum age of reproduction: 15 yr - Maximum number of progeny per year: 10 offspring - Sex ratio at birth: 50% females and 50% males | [2] |
| Reproductive rates | - Percent of females breeding: 7 scenarios (100, 90, 80, 70, 60, 50, and 40%) to account for nest predation - Environmental variation in breeding: 0.5 - Offspring per female: the largest productivity per female in absence of nest predation (5.52) assigned to the population with the greatest juvenile to adult proportion, the values of other populations rescaled to their relative proportions with respect to the maximum. - Standard Deviations: estimated over three years of study | [2],[3], This study |
| Mortality rates | - Males and females across all ages: 54 % ± 1.37 SD | This study |
| Initial population size | - From mapping male territories | [4] |
| Carrying capacity | - Maximum density observed in a population (4 individuals/10 ha) multiplied for patch size | [1] |
| Mate monopolization | - None (100% males breeding) | (*) |
| Inbreeding depression | - None | (*) |
| Catastrophes | - None | (*) |

(*) Very conservative simulations were run, supposing no catastrophes nor inbreeding depression occurring, and all males reproducing.

**References**

1. Laiolo P, Tella JL (2007) Erosion of animal cultures in fragmented landscapes. Front Ecol Environ 5: 68-72
2. Cramp S (1988) The Birds of the Western Palearctic. Vol. 5. Oxford Univ. Press, New York
3. Yanes M, Suarez F (1996) Incidental nest predation and lark conservation in an Iberian semiarid shrubsteppe. Conservation Biology 10: 881-887
4. Tella JL, Vögeli M, Serrano D, Carrete M (2005) Current status of the endangered Dupont’s lark in Spain: overestimation, decline, and extinction of local populations. Oryx 39: 90-94
